# Comparing the prevalence of *Helicobacter pylori* and virulence factors *cagA*, *vacA, *and *dupA* in supra-gingival dental plaques of children with and without dental caries: a case–control study

**DOI:** 10.1186/s12903-022-02175-5

**Published:** 2022-05-09

**Authors:** Aida Mehdipour, Parisa Chaboki, Farzaneh Rasouli Asl, Mohammad Aghaali, Negar Sharifinejad, Saeed Shams

**Affiliations:** 1 Department of Pediatric Dentistry, Faculty of Dentistry, Qom University of Medical Sciences, Qom, Iran; 2 Student Research Committee, Qom University of Medical Sciences, Qom, Iran; 3 Cellular and Molecular Research Center, Qom University of Medical Sciences, Qom, Iran; 4 Department of Epidemiology, School of Medicine, Qom University of Medical Sciences, Qom, Iran

**Keywords:** *Helicobacter pylori*, Children, Dental plaque, Dental caries, Dental decay, DMFT, Dmft, PCR, Virulence genes, *cagA*, *vacA*, *dupA*

## Abstract

**Background:**

*Helicobacter pylori* infection is one of the most common infectious diseases in humans. Dental plaque is considered as a reservoir of this bacterium, which could play an important role in the development of gastrointestinal problems. Our aim was to investigate the prevalence of *H. pylori* and its virulence factors in dental plaques in children with and without dental caries.

**Methods:**

Among children aged 6 to 12 years, a total of 72 children were enrolled in the study, including 36 cases with total DMFT/dmft > 3 (case group) and 36 participants with total DMFT/dmft < 1 (control group). After removing supra-gingival plaques from the lower first permanent molar teeth, the samples were examined using PCR method for the presence of *H. pylori* and some of its virulence factors. Statistical analysis was performed using chi-square, Fisher' exact test, t-tests, and logistic regression.

**Results:**

Of 72 participants, 40 cases were male, and 32 cases were female. The minimum and maximum values of total DMFT/dmft indices were zero and ten, respectively, and the mean ± SD value of total DMFT/dmft was 2.78 ± 3.22. Except for vegetable consumption (*p* = 0.045), there was no significant difference between the two groups regarding gastrointestinal disorders, feeding methods in infancy (*p* = 0.058), frequency of daily brushing (*p* = 0.808), frequency of dental visits (*p* = 0.101), and history of dental scaling (*p* = 0.246) and professional topical fluoride therapy (*p* = 0.5). Out of 72 samples, 15 cases were positive for *H. pylori* DNA (20.8%), and there was no significant association between the presence of this bacterium in dental plaque and dental caries (*p* = 0.281). The frequency of virulence factors detected in 15 *H. pylori* cases was as follows: *cagA* in six cases (40.0%), *vacAm1* in three cases (20.0%), and *vacAs1* in one case (6.7%). There was no significant difference between the groups regarding the prevalence of virulence factors.

**Conclusion:**

Our results indicate the presence of *H. pylori* along with some virulence factors in dental plaques as a reservoir of this bacterium in children in Iran. Although there was no significant association between this bacterium and the incidence of dental caries, dental health in children needs to be seriously taken into consideration.

## Introduction

*Helicobacter pylori* is a Gram-negative, spiral-shaped, motile bacterium that colonizes gastric epithelial cells. Infection by this bacterium is a global public health problem that affects more than 50.0% of the world's population [1, 2]. It has been shown that *H. pylori* is mainly acquired in childhood, and its incidence and prevalence vary in different regions [3]. In developed countries with high annual incomes and high levels of health services, the prevalence rate of *H. pylori* infection among children has been reported to be 1.8 to 65.0%. In middle and low-income countries with high population and low quality of health, the prevalence of this bacterium is higher than the global rate [4, 5]. Although the exact age at which children become infected with the bacterium is unknown, the findings suggest that the colonization occurs before the age of 5. This infection seems to be contagious, especially in developing countries, because it is found in all family members [6, 7].

In most individuals, *H. pylori* colonization in the stomach does not cause symptoms. Nevertheless, the prolonged presence of the bacterium can be associated with various gastrointestinal diseases, including gastric ulcer (GU), duodenal ulcer (DU), gastritis, gastric cancer (GC), and MALT lymphoma. Based on other studies, about < 3.0% and < 0.1% of people suffering from *H. pylori* infection usually develop gastric cancer and MALT lymphoma, respectively [8–10].

Evidence suggests that oral cavity temperature, pH, and microaerophilic conditions are suitable for *H. pylori* colonization, especially in dental biofilm [11, 12]. Therefore, simultaneous colonization has been reported in dental plaques and gastrointestinal biopsies [13]. However, the presence of this bacterium in dental plaque is associated with poor oral hygiene and could increase the risk of recurrence of gastrointestinal problems [14]. Studies have also reported the incidence of *H. pylori*-associated halitosis, glossitis, aphthous stomatitis, and dental decay [15].

According to different studies, a high level of genetic diversity has been documented in the virulence genes of *H. pylori* in different geographic areas, which can potentially contribute to the pathogenesis of infections caused by this microorganism [16, 17]. Among virulence factors, CagA and VacA are important pathogenic oncoproteins involved in colonization, damage to the gastric epithelium, increased cell proliferation, chronic inflammation, and carcinogenic processes. Activation of the intracellular signaling pathway and the resulting intracellular changes increase the risk of gastric cancer by inducing the activation of the NFκB signaling pathway and increasing IL-8 levels [18, 19]. The DU-promoting gene (*dupA*), located in the plasticity region of the *H. pylori* genome, was initially described as a risk marker for the development of DU and as a protective factor against GC [16]. Conversely, some evidence has also been suggested that this factor is associated with stomach cancer [20]. The selection of virulence factors is significant to determine the disease risk due to microbial agents. The pathogenic role of *cagA*, *vacA*, and *dupA* genes in various diseases caused by *H. pylori* in different age and sex groups has been proven worldwide [21, 22].

According to the World Health Organization (WHO), gastric cancer accounted for 16.4% of all cancer-related deaths in Iran in 2020 (http://gco.iarc.fr—access date to the website: 02.01.2022), indicating the importance of its early diagnosis and prevention. However, in our country, more than 80.0% of the population aged > 40 years has a history of *H. pylori* infection, which can be considered one of the leading causes of GC [23]. Certainly, one of the most important ways to prevent *H. pylori*-associated gastrointestinal diseases is to reduce its colonization in childhood, which leads to a reduction in the rate of infection, inflammation, and various diseases because *H. pylori* infection is usually acquired early in life [24]. Therefore, due to the lack of sufficient information and evidence in this field and the scarcity of studies comparing the prevalence of pathogenic strains of *H. pylori* in dental plaques in patients with and without dental caries, especially in children, this study was conducted for the first time in Iran to investigate the prevalence of *H. pylori* and its different genotypes in dental plaques collected from children aged 6–12 years.

## Methods

### Patients and sampling

This case–control study was conducted from 2020 to 2021 on children referred to the dental clinic of Qom University of Medical Sciences, Qom, Iran. The sample size was determined to be 36 people in each of the case and control groups based on a study by Silva et al. [25] and by considering the prevalence rates of 0.03 and 0.25% for *H. pylori* with a type I error of 0.05 and power of 0.8. Random sampling method was used to select children in the age range of 6 to 12 years. The case group consisted of 36 children with total DMFT/dmft > 3, and the control group consisted of 36 caries-free children with DMFT/dmft < 1. Two groups were matched based on age and sex.

Exclusion criteria for excluding children from the study were as follows: antibiotic use during the two weeks prior to sampling, lack of lower first permanent molar tooth, and reluctance of children and/or their parents/legal guardians to cooperate in this study for any reason. The required information was collected through a data collection form provided to the individuals mentioned above, including patient demographic information, symptom or history of gastrointestinal diseases, history of gastrointestinal medications use, frequency of fast food consumption, frequency of dairy and vegetable consumption, frequency of daily tooth brushing, frequency of dental visits, and history of dental scaling and professional topical fluoride therapy.

Supra-gingival plaque samples were collected from the lower first permanent molars in both groups with the help of a catheter and other manual scaling instruments (Hu-Friedy, Chicago, IL, USA). Each collected plaque sample was transferred into a microtube containing 0.5 mL of sterile distilled water and stored in a freezer at − 20 °C until used for testing.

### DNA extraction

The extraction of the genome was performed using a commercial kit (Fovergen, Taiwan) according to the manufacturer's instructions, and the quality of the DNA extracted from the samples was evaluated using a NanoDrop spectrophotometer (Thermo Fisher Scientific, USA).

### Detection of *H. pylori *and virulence genes using PCR

Amplification of the 16S rRNA fragment was performed using the PCR technique to detect *H. pylori* DNA in dental plaque samples. In the next step, all positive samples containing the 16S rRNA gene were subjected to confirmatory analysis for the presence of virulence genes *cagA*, *vacAm1, vacAm2*, *vacAs1/s2*, and *dupA*. PCR reactions were performed with a final volume of 25 μL, containing 9 μL of Master Mix 2X (Ampliqon, Denmark), 1 μL of each primer (Metabion, Germany) (10 pmol/ μl) (Table 1), 4 μL of extracted genome (100 ng), and 10 μL of sterile DW. PCR amplification thermal cycling conditions were as follows: an initial genome denaturation step at 95 °C for 5 min (one cycle), followed by 33 cycles of denaturation at 94 °C for 30 s, primer annealing at 54 °C for 60 s, and extension at 72 °C for 30 s, and then a final extension step at 72 °C for 5 min. The genome extracted from the reference strain of *H. pylori* J99 was used as a positive control. The PCR product was electrophoresed with 1.0% agarose gel and examined by ultraviolet light.

**Table 1:** Primers used in this study

Target Gene	Sequence (5' → 3')	Size of products (bp)	Reference
16S rRNA	F: CTGGAGAGACTAAGCCCTCC R: ATTACTGACGCTGATTGTGC	110	[26]
*cagA*	F: CGGTATCAGTGGCTAAAGC R: AGCAACTTGAGCGTAAATG	377	[27]
*dupA*	F: GACGATTGAGCGATGGGAATAT R: CTGAGAAGCCTTATTATCTTGTTGG	971	[21]
*vacAm1*	F: GGTCAAAATGCGGTCATGG R: CCATTGGTACCTGTAGAAAC	290	
*vacAm2*	F: GGAGCCC*CAG*GAAACATTG R: CATAACTAGCGCCTTGCAC	352	
*vacAs1/s2*	F: ATGGAAATACAACAAACACAC R: CTGCTTGAATGCGCCAAAC	259/286	

### Statistical analysis

Statistical analysis was performed using SPSS statistics software version 22 (IBM, NY, USA) by employing chi-square, Fisher' exact test, t-tests, and logistic regression to compare the prevalence of *H. pylori* cases between the two groups. A *p*-value of less than 0.05 was considered as statistically significant.

## Results

Among 72 participants, 40 (55.6%) cases were male, and 32 (44.4%) cases were female, with a mean ± SD age of 7.97 ± 1.83 years. The minimum and maximum values of total DMFT/dmft were zero and ten, respectively, and the mean ± SD value of total DMFT/dmft was 2.78 ± 3.22.

Fifty-nine participants under study (81.9%) had no gastrointestinal symptoms/diseases, only one participant had two symptoms simultaneously, and the rest had one symptom or history of gastrointestinal disease. Symptoms/diseases were as follows: stomach pain (6.9%), gastroesophageal reflux (5.6%), gastric ulcer (2.8%), constipation (2.8%), and gastritis (1.4%). The average frequency of fast food consumption was 2 times a month (ranging from 1 to 12 times). Most children consumed dairy products and vegetables 4 times a week (62.5 and 56.9%, respectively). In terms of nutritional status in infancy, 83.3 and 16.7% of children consumed breast milk and formula, respectively. Also, 5.6% of children referred to the dentist once every few years, 54.2% every year, 11.0% every six months, 1.4% every three months, and 27.8% only during pain. In addition, 4.2% of the study population had a history of professional topical fluoride therapy, and 2.8% had a history of dental scaling. The average frequency of daily tooth brushing was 0.83, ranging from 0 to 2 times per day.

According to the results, there was no significant difference between the two groups in terms of gastrointestinal disorders, feeding methods in infancy, and frequency of fast food and dairy consumption (*p* > 0.05). More information is provided in Table 2. The results of logistic regression analysis showed that the low vegetable consumption variable could be associated with tooth decay, and the odds ratio of caries in children with low vegetable consumption compared to those with high vegetable consumption was 2.9 (*p* = 0.045). More information is presented in Table 3.

**Table 2:** Frequency distribution of demographic variables and factors affecting dental decay in the two groups

Variable	Groups			
	Total N (%)	Case N (%)	Control N (%)	*p* value
Age (mean ± SD)	7.97 ± 1.83	7.83 ± 1.96	8.11 ± 1.7	0.524
Sex (Boy)	40 (55.6)	20 (55.6)	20 (55.6)	-
Gastric ulcer	2 (2.8)	0	2(5.6)	0.246
GE reflux	4 (5.6)	3 (8.3)	1 (2.8)	0.307
Constipation	2 (2.8)	1 (2.8)	1 (2.8)	-
Gastritis	1 (1.4)	0	1 (2.8)	0.5
Consumption of dairy products				
Low	7 (9.7)	5 (13.9)	2(5.6)	0.193
Medium	45 (62.5)	24 (66.7)	21 (58.3)	
High	20 (27.8)	7 (19.4)	13 (36.1)	
Consumption of vegetable				
Low	6 (8.3)	4(11.1)	2(5.6)	0.198
Medium	41(56.9)	23(63.9)	18(50.0)	
High	25(34.7)	9(25.0)	16(44.4)	
Feeding methods in infancy				
Breastfeeding	60 (83.3)	33 (91.7)	27 (75.0)	0.058
Formula feeding	12 (16.7)	3 (8.3)	9 (25.0)

**Table 3:** Logistic regression analysis of predictors of dental decay

Variable	Beta	Standard Error	*p*-value	OR (95.0% C.I)
Step 1				
Age	−0.13	0.15	0.393	0.88 (0.66–1.18)
Consumption of dairy (High)			0.501	
Consumption of dairy (Medium)	1.28	1.09	0.240	3.60 (0.43–30.39)
Consumption of dairy (Low)	0.42	0.66	0.522	1.53 (0.42–5.59)
Low consumption of vegetable	0.83	0.64	0.191	2.30 (0.66–8.05)
Frequency of fast food consumption every month	0.07	0.13	0.608	1.07 (0.83–1.38)
Frequency of daily tooth brushing	0.22	0.68	0.743	1.25 (0.33–4.73)
Fluoride therapy	0.73	1.31	0.579	2.06 (0.16–26.66)
Constant	−0.31	1.48	0.832	0.73

Step 6				
Low consumption of vegetable	1.09	0.54	0.045	2.96 (1.03–8.53)
Constant	−0.76	0.46	0.096	0.47

There was no significant difference between the two groups in terms of frequency of dental visits (*p* = 0.101), frequency of daily tooth brushing (*p* = 0.808), and history of professional topical fluoride therapy (*p* = 0.5) and dental scaling (*p* = 0.246).

PCR detection of the 16S rRNA gene showed that 15 out of 72 dental plaque samples contained *H. pylori* (20.8%). There was no statistically significant difference between the case (9 of 36 cases-25.0%) and control (6 of 36 cases-16.7%) groups in terms of the presence of *H. pylori* in dental plaques (*p* = 0.281). The prevalence of *H. pylori* DNA in dental plaques was 22.5% in boys and 18.8% in girls (*p* = 0.697). Out of 15 *H. pylori*-positive plaques, only 10 cases (66.7%) were also positive for the virulence genes. Among, 6 (40.0%) samples were *cagA* positive, of which three samples belonged to the case group, and three samples belonged to the control group (*p* = 0.455). Also, 3 (20.0%) and 1 (6.7%) plaques were positive for the presence of *vacAm1* and *vacAs1* genes, respectively, all belonged to the case group. None of the samples contained *vacAm2* and *dupA* virulence genes (Table 4). Moreover, one of the ten positive samples for virulence genes in the case group had *cagA* + *vacAm1* + *vacAs1* genotypes simultaneously. No significant difference was observed between the two groups regarding virulence factors.

**Table 4:** Frequency of virulence genes in case and control groups

Genes	Groups		
	Total N (%)	Case N (%)	Control N (%)
16S rRNA	15 (20.8)	9 (60.0)	6 (40.0)
*cagA*	6 (40.0)	3 (50.0)	3 (50.0)
*dupA*	0	0	0
*vacAm1*	3 (20.0)	3 (100.0)	0
*vacAm2*	0	0	0
*vacAs1*	1 (6.7)	1 (100.0)	0

## Discussion

In some cases, antibiotic treatment often fails to treat *H. pylori* infection, suggesting that there may be other places where the organism could survive and cause recurrence. Dental plaques could be considered as an important potential reservoir for bacterial re-colonization of the stomach [13, 28]. Therefore, this study was conducted for the first time in Iran to investigate the prevalence of *H. pylori* and its important virulence genes in dental plaques collected from children aged 6–12 years.

According to the results, there was no significant difference between the case and control groups regarding gastrointestinal symptoms/diseases reported by participants, including gastric ulcer, gastric reflux, constipation, and gastritis. In a systematic review study by Lechien et al. (2020), no significant association was found between gastric reflux and dental problems, which is consistent with the present study results [29].

In the current study, there was no significant difference in the frequency of fast food consumption between the two groups, and most subjects consumed fast food once a month. The frequency of dairy consumption in most subjects was reported to be 4 times a week, and there was no significant difference between the case and control groups in terms of the consumption of dairy products. Nadelman et al. conducted a review study to investigate the effect of probiotic dairy products on teeth. Their results showed that consumption of dairy products, especially yogurt and milk, was effective in reducing the colonization of *Streptococcus mutans*, increasing salivary pH, and increasing plaque index [30]. This finding is inconsistent with the present study results, which may be due to differences in the type of dairy products consumed by subjects in these two reports.

In this study, there was a significant association between vegetable consumption and dental caries. In line with this study's results, Yen et al. found that people who consumed more vegetables were significantly less likely to develop dental decay [31]. In another study on Japanese children, the habit of consuming vegetables at the beginning of a meal (Vege-first) was found to be associated with the prevention of tooth decay [32].

According to the results, there was no significant difference between the case and control groups in terms of dental care, including frequency of dental visits, frequency of daily tooth brushing, and history of professional topical fluoride therapy and dental scaling. In a study by El Batawi et al. (2020), no significant association was found between the frequency of tooth brushing and the presence of *H. pylori* in children [11]. The lack of an association between the two seems to be related to brushing at irregular intervals, resulting in their inefficiency and ineffectiveness in preventing caries. However, the quality of brushing is much more effective in making quantitative and qualitative changes in dental plaques compared to the frequency of daily brushing.

There are several methods used to detect *H. pylori*, including culture, urease tests, histopathology, serological methods, and PCR techniques. In this study, the PCR method was used to detect *H. pylori* and its virulence factors, which is more specific than the other tests [33, 34]. Overall, the prevalence rate of *H. pylori* DNA in dental plaque samples was 20.8%, and no significant difference was observed between the two groups. However, the higher prevalence of these bacteria in the case group than in the control group was considerable. Studies have shown that the prevalence of *H pylori* in dental plaques varies from 0.0 to 100.0% [35]. For example, *H. pylori* DNA was detected in 72.0% of dental plaque samples examined in a study by Assumpção et al. in Brazil [36]. In other studies by Eskandari et al., Momtaz et al., and Tahbaz et al. in adults in Iran, *H. pylori* genome was identified in 5.9%, 0.0%, and 5.0% of dental plaques, respectively [37–39]. In a 2000 study conducted in Germany, 41 dental plaque samples (97%) were positive for *H. pylori* [40].

In this study, no significant association was found between the presence of *H. pylori* in dental plaque and the incidence of dental caries. In contrast, in a study by Liu et al. in adults, a significant association was found between *H. pylori* colonization within the oral cavity and dental caries and poor dental hygiene [41]. El Batawi et al. also detected *H. pylori* in 30.0% of dental biofilm samples of children aged 4–7 years with severe caries lesions, and there was a significant difference in dmft index between children with *H. pylori*-positive and *H. pylori*-negative dental biofilm samples [11]. This difference in results between different reports could be attributed to differences in the age range of populations studied, which may affect the rate of bacterial colonization.

Regarding the prevalence of virulence genes, the *cagA* gene was detected in 40.0% of the samples in both groups. This result is similar to our previous study in Qom in 2019 [27], in which the *cagA* gene was detected in 32.5% (13 cases) and 27.5% (11 cases) of patients with gastritis and gastric cancer, respectively. Other studies have also reported different prevalence rates for this gene. In a study conducted in Iran, the *cagA* gene was detected in 4 (3 in periodontitis group and one in non-periodontitis group) out of 5 specimens of *H. pylori*-positive dental plaque [39]. In two studies in Mexico, Flores-Treviño et al. (2019) reported that 21.7% of *H. pylori*-positive dental plaque samples contained the *cagA* gene [42], while Mendoza-Cantú et al. (2017) indicated this gene in 71.1% of dental plaques collected from asymptomatic Mexican children [43]. The *vacAs1* (one case) and *vacAm1* (three cases) genes were detected only in the case group; however, no significant difference was found between the two groups in terms of the presence of these virulence genes. In addition, none of the samples contained *dupA* and *vacAm2* genes. In other studies, the prevalence of these genes has also been reported to be low. In a study by Rasmussen et al. (2012) in São Paulo, Brazil, only 14 strains of *H. pylori* isolated from dental plaques harbored *vacAs1*, while none of the isolates harbored *vacAm1*and *vacAm2* [44]. Tahbaz et al. also identified *vacAs1*, *vacAm1*, and *vacAm2* in 3 (3.0%), 3 (3.0%), and 2 (2.0%) samples of *H. pylori-*positive dental plaques, respectively [39].

## Conclusion

This research is the first study demonstrating the presence of *H. pylori* and some virulence genes in dental plaques as a potential reservoir of this bacterium in children in Iran. Although in this project, no significant association was found between the presence of this bacterium and its virulence factors in dental plaque and the incidence of dental caries, *H. pylori* colonization in the mouth should not be ignored. However, *H. pylori* strains have a high potential for inducing gastrointestinal diseases, and one of the best ways to prevent serious diseases caused by this bacterium is to observe oral and dental hygiene. In addition, our findings indicated the low frequency of virulence genes among the strains under study, which may be due to their low pathogenicity in our geographical area. However, further studies are recommended to more accurately evaluate this finding and also to evaluate other virulence genes.

### Limitations

Due to financial and time constraints, we were unable to study other virulence genes as well as their expression.

## Data Availability

The datasets generated and/or analysed during the current study are not publicly available due to the subject matter specialization, but are available from the corresponding author on reasonable request.

## Abbreviations

DMFT/dmft: Decayed, missing, and filled teeth
PCR: Polymerase chain reaction
16S rRNA: 16S ribosomal ribonucleic acid
DNA: Deoxyribonucleic acid
MALT: Mucosa-associated lymphoid tissue
GC: Gastric cancer
IL-8: Interleukin-8
*cagA*: Cytotoxin-associated gene A
*vacA*: Vacuolating cytotoxin gene A
*dupA*: Duodenal ulcer promoting gene A
NF-κB: Nuclear factor kappa B
DW: Distilled water
GE reflux: Gastroesophageal reflux
SD: Standard deviation
bp: Base pair
